# Extracorporeal Membrane Oxygenation Modulates the Inflammatory Milieu and Organ Failure Trajectory in Severe COVID-19 and Sepsis

**DOI:** 10.3390/jcm14124224

**Published:** 2025-06-13

**Authors:** Nicoleta Barbura, Tamara Mirela Porosnicu, Cristian Oancea, Dorel Sandesc, Marius Papurica, Ovidiu Bedreag, Ciprian Gîndac, Adelina Raluca Marinescu, Ruxandra Laza, Voichita Elena Lazureanu

**Affiliations:** 1 Doctoral School, “Victor Babes” University of Medicine and Pharmacy, 300041 Timisoara, Romania; nicoleta.marian@umt.ro; 2 Anaesthesia and Intensive Care Research Center, Faculty of Medicine, “Victor Babes” University of Medicine and Pharmacy, 300041 Timisoara, Romania; marius.papurica@umft.ro (M.P.); bedreag.ovidiu@umft.ro (O.B.); ciprian.gindac@umft.ro (C.G.); 3 Center for Research and Innovation in Precision Medicine of Respiratory Diseases (CRIPMRD), “Victor Babes” University of Medicine and Pharmacy, 300041 Timisoara, Romania; oancea@umft.ro; 4 Discipline of Infectious Disease, “Victor Babes” University of Medicine and Pharmacy, 300041 Timisoara, Romania; adelina.marinescu@umft.ro (A.R.M.); laza.ruxandra@umft.ro (R.L.); lazureanu.voichita@umft.ro (V.E.L.)

**Keywords:** COVID-19, extracorporeal membrane oxygenation, cytokines, Severity of Illness Index, multiple organ failure

## Abstract

**Background and Objectives:** Coronavirus disease 2019 (COVID-19) triggers a dysregulated host response that may culminate in refractory hypoxaemic shock. Whether veno-venous ECMO modifies the inflammatory cascade more effectively in COVID-19 than in other septic states, and how it compares with conventional ventilatory support for COVID-19, remains uncertain. We compared three groups: COVID-19 patients supported with ECMO (COVID-ECMO, n = 25), non-COVID-19 septic shock patients on ECMO (SEPSIS-ECMO, n = 19) and critically ill COVID-19 patients managed without ECMO (COVID-CONV, n = 74). **Methods:** This retrospective study (January 2018–January 2025) extracted demographic, laboratory and clinical data at baseline, 48 h and 72 h. The primary end-point was the 72 h change in SOFA score (ΔSOFA). The secondary end-points included the evolution of interleukin-6 (IL-6), C-reactive protein (CRP), D-dimer and ferritin; haemodynamic variables; and 28 day mortality. A post hoc inverse-probability-of-treatment weighting (IPTW) sensitivity analysis adjusted for between-group severity imbalances. **Results**: Baseline APACHE II differed significantly (29.5 ± 5.8 COVID-ECMO, 27.4 ± 6.1 SEPSIS-ECMO, 18.2 ± 4.9 COVID-CONV; *p* < 0.001). At 48 h, IL-6 fell by 51.8% in COVID-ECMO (−1 116 ± 473 pg mL^−1^) versus 32.4% in SEPSIS-ECMO and 18.7% in COVID-CONV (*p* < 0.001). The ΔSOFA values at 72 h were −4.6 ± 2.2, −3.1 ± 2.5 and −1.4 ± 1.9, respectively (*p* < 0.001). ECMO groups achieved larger mean arterial pressure rises (+16.8 and +14.2 mmHg) and greater norepinephrine reduction than COVID-CONV. The twenty-eight-day mortality was 36.0% (COVID-ECMO), 42.1% (SEPSIS-ECMO) and 39.2% (COVID-CONV) (*p* = 0.88). Across all patients, IL-6 clearance correlated with ΔSOFA (ρ = 0.48, *p* < 0.001) and with vasopressor-free days (ρ = 0.37, *p* = 0.002). **Conclusions**: ECMO, regardless of aetiology, accelerates inflammatory-marker decline and organ failure recovery compared with conventional COVID-19 management, but survival advantage remains elusive. COVID-19 appears to display a steeper cytokine-response curve to ECMO than bacterial sepsis, suggesting phenotype-specific benefits that merit confirmation in prospective trials.

## 1. Introduction

Septic shock and coronavirus disease 2019 (COVID-19) share a final common pathway of uncontrolled systemic inflammation that erodes micro- and macro-circulatory integrity, precipitating multi-organ failure [1,2,3]. Worldwide, sepsis is responsible for ≈11 million deaths each year, and contemporary adult veno-venous extracorporeal membrane oxygenation (VV-ECMO) registry data place intensive care mortality between 40% and 60% once conventional ventilation or shock therapy fails [4,5].

Although both entities are cytokine-driven, COVID-19-associated acute respiratory distress syndrome (ARDS) is distinguished by diffuse endothelialitis, immunothrombosis and prolonged viral replication, whereas bacterial sepsis follows a more biphasic hyper-/hypo-inflammatory trajectory [1,2,6]. The Extracorporeal Life Support Organization (ELSO) observed a time-dependent decline in COVID-19 ECMO survival from 62% in early 2020 to 46% by late 2020 [6,7], and meta-omic analyses demonstrate higher D-dimer and ferritin peaks in viral ARDS, while bacterial sepsis shows earlier monocyte HLA-DR exhaustion and lymphocyte anergy [8,9].

Early reductions in inflammatory markers correlate with outcome. In a 191-patient cohort, a ≥50% fall in C-reactive protein (CRP) by 72 h carried an adjusted odds ratio of 0.31 for ICU mortality [10]. Similarly, a single-centre study of 244 COVID-19 admissions found D-dimer to be the most sensitive (96%) and specific (92%) predictor of severe disease, independent of age and comorbidity [11]. The updated Sequential Organ Failure Assessment (SOFA) framework now emphasises these dynamic surrogates, reflecting a mechanistic shift from static scoring towards trajectory-based risk assessment [12].

Adjunctive extracorporeal blood-purification strategies aim to temper cytokinaemia. A narrative review highlighted the immunomodulatory benefits of high-volume haemofiltration and adsorption in septic shock, albeit with heterogeneous evidence [13]. Case reports of ECMO-integrated CytoSorb^®^ have documented brisk interleukin-6 clearance and catecholamine sparing [14], and the multi-centre CytoSorb Therapy in COVID-19 Registry reported 74% 90-day survival when adsorption was initiated within 24 h of cannulation [15]. A 2025 systematic review of 744 septic shock patients confirmed a pooled odds ratio of 0.49 for 28-day mortality with CytoSorb^®^ therapy [16], findings supported by experimental data showing reduced renal NF-κB activation in ECMO + CRRT porcine models [17], and a 2019 propensity-weighted analysis demonstrated lower observed-versus-expected mortality in classical sepsis [18]. Beyond purification, comparative outcome studies suggest an intrinsic survival benefit of ECMO. A propensity-matched analysis of 98 patients with severe COVID-19 respiratory failure showed an absolute 18% reduction in in-hospital mortality for ECMO versus maximal ventilation alone [19,20]. Such data underscore the need for granular, phenotype-specific evaluation rather than extrapolation from heterogeneous historical cohorts.

Building on these gaps, we designed a study with the following objectives: (i) to compare inflammatory-marker trajectories between COVID-19 and non-COVID-19 patients on ECMO; (ii) to contrast ECMO-treated COVID-19 with conventionally managed severe COVID-19. We hypothesise that ECMO accelerates biomarker decline and SOFA improvement, with the steepest response in COVID-ECMO patients.

## 2. Materials and Methods

### 2.1. Study Design and Setting

A multi-centre study was conducted between January 2018 and January 2025 at the Victor Babes Hospital of Infectious Diseases and “Pius Brinzeu” Clinical Emergency Hospital, affiliated with the “Victor Babeș” University of Medicine and Pharmacy, Timișoara. The Local Commission of Ethics for Scientific Research operates under article 167 provisions of Law no. 95/2006, art. 28, chapter VIII of order 904/2006; within EU GCP Directive 2005/28/EC, International Conference of Harmonisation of Technical Requirements for Registration of Pharmaceuticals for Human Use (ICH); and within the Declaration of Helsinki—Recommendations Guiding Medical Doctors in Biomedical Research Involving Human Subjects.

### 2.2. Cohort Definition and Eligibility Criteria

Patients were divided a priori into three mutually exclusive groups. The first group, COVID-ECMO (n = 25), included patients with reverse transcriptase PCR–confirmed SARS-CoV-2 infection who underwent veno-venous ECMO initiated due to refractory hypoxaemia or hypercapnia despite optimised mechanical ventilation. The second group, SEPSIS-ECMO (n = 19), comprised patients with septic shock unrelated to COVID-19, meeting the Sepsis-3 criteria—suspected or confirmed infection, serum lactate levels exceeding 2 mmol L^−1^ and a vasopressor requirement to maintain a mean arterial pressure of ≥65 mmHg—and who required veno-venous ECMO for severe respiratory failure. The third group, COVID-CONV (n = 74), consisted of critically ill COVID-19 patients classified as being in World Health Organization severity categories 5–7 (defined by PaO_2_/FiO_2_ ratios below 150 mmHg or the need for vasopressors or renal replacement therapy), managed without ECMO throughout their ICU admission.

ECMO initiation criteria were uniform across centres:

- PaO_2_/FiO_2_ < 80 mmHg for >6 h or <50 mmHg for >3 h despite optimisation;
- pH < 7.20 with PaCO_2_ > 85 mmHg for >6 h;
- Refractory shock with norepinephrine > 0.5 µg kg^−1^ min^−1^ for >2 h.

All patients across these groups were required to meet specific inclusion criteria: age ≥18 years, availability of two paired inflammatory-marker panels (obtained at baseline and again after 48 h) and complete daily Sequential Organ Failure Assessment (SOFA) scores documented for the initial 72 h. Of 214 screened candidates, 96 were excluded for the following reasons: chronic dialysis/CKD-5 (n = 12), veno-arterial or hybrid ECMO (n = 23), ECMO < 24 h (n = 11), incomplete IL-6 data (n = 28) and missing 28-day status (n = 22). A sensitivity analysis that excluded seven patients cannulated >48 h after meeting WHO-critical criteria reproduced all the significant findings.

The study applied exclusion criteria as well, which encompassed patients who were chronically dialysis-dependent or had stage 5 chronic kidney disease (estimated glomerular filtration rate below 15 mL min^−1^ per 1.73 m^2^), those receiving ECMO support for less than 24 h due to circumstances such as emergency withdrawal due to futility, patients on concomitant veno-arterial or hybrid ECMO configurations and those with missing essential outcome data, including 28 day survival status or ICU length of stay.

To minimise immortal-time bias, the index time-point (T_0_) was uniformly established for all participants as either the hour of ECMO cannulation for patients receiving ECMO support or the time-point at which WHO-critical criteria were first met for patients with COVID-19 managed conventionally without ECMO. The median admission to T_0_ intervals were 42 h (IQR 31–54) in the COVID-ECMO group, 36 h (25–47) in the SEPSIS-ECMO group and 11 h (8–17) in the COVID-CONV group. A sensitivity analysis that excluded seven patients cannulated >48 h after meeting WHO-critical criteria reproduced all the significant findings.

### 2.3. Treatment Protocols

Veno-venous ECMO was implemented using femoro-jugular cannulation and a centrifugal pump/polymethylpentene membrane oxygenator. The target blood flow was 4.0–4.5 L min^−1^ with a sweep gas flow titrated to achieve PaCO_2_ 35–45 mmHg. Ventilator settings were switched to ultra-protective mode (tidal volume 3–4 mL kg^−1^ predicted body weight, PEEP 12–15 cmH_2_O, driving pressure ≤ 12 cmH_2_O).

All ECMO circuits incorporated a high-cut-off polyethersulfone haemofilter. Replacement fluid was delivered in mixed pre-/post-dilution forms with a target effluent dose of 28–35 mL kg^−1^ h^−1^ (standard) or ≥45 mL kg^−1^ h^−1^ (high-dose) according to attending physician discretion. Anticoagulation used unfractionated heparin, titrated to anti-Xa 0.30–0.50 IU mL^−1^ or activated partial thromboplastin time 55–70 s; bolus-free initiation was favoured unless pre-existing clot burden warranted loading. Membrane exchange was mandated when transmembrane pressure exceeded 250 mmHg or filtration fraction surpassed 30%. No stand-alone haemoadsorption device (e.g., CytoSorb^®^) was used in any patient.

COVID-CONV patients received the guideline-directed standard of care, including the following: (i) Volume-controlled ventilation (6 mL kg^−1^ PBW), rescue prone positioning, neuromuscular blockade for PaO_2_/FiO_2_ < 100 mmHg and inhaled nitric oxide as needed. (ii) Pharmacotherapy: intravenous dexamethasone 6 mg day^−1^ × 10 days, remdesivir 100 mg day^−1^ × 5 days when symptom onset ≤ 10 days and a single 8 mg kg^−1^ dose of tocilizumab if CRP > 75 mg L^−1^ with escalating oxygen demand. (iii) Renal replacement therapy (flexible continuous veno-venous haemodiafiltration at 30 mL kg^−1^ h^−1^) when conventional indications arose (KDIGO stage 3 AKI, volume overload, or hyperkalaemia). All COVID-ECMO patients received the identical dexamethasone–remdesivir–tocilizumab bundle unless contraindicated. RRT was used in 17/25 (68%) of COVID-ECMO, 14/19 (74%) of SEPSIS-ECMO and 21/74 (28%) of COVID-CONV patients; RRT exposure was forced into all multivariable models.

### 2.4. Data Acquisition and Variables

Continuous variables displaying normal distribution were summarised as means ± SD, and demographic data including age, sex and body mass index (BMI), along with comorbidities (as captured by components of the Charlson index), infection sources, microbiological findings and time to antibiotic administration were collected. Severity was assessed using the Acute Physiology and Chronic Health Evaluation II (APACHE II) score during the initial 24 h, daily Sequential Organ Failure Assessment (SOFA) scores throughout the first 72 h and the WHO Ordinal Scale. The respiratory parameters gathered included the ratio of arterial partial pressure of oxygen to fractional inspired oxygen (PaO_2_/FiO_2_), the static compliance of the respiratory system and ventilatory ratio. Haemodynamic monitoring encompassed mean arterial pressure (MAP), heart rate and vasopressor dosage, standardised as norepinephrine equivalents.

IL-6 samples were obtained at −1 h (immediately before ECMO cannulation or WHO criteria fulfilment), +6 h, +48 h and +72 h. The median time from hospital admission to T0 was 42 h for COVID-ECMO, 36 for SEPSIS-ECMO and 11 h for COVID-CONV.

Inflammatory mediators were quantified using validated laboratory techniques in the hospital′s core laboratory. Interleukin-6 (IL-6) levels were determined by electrochemiluminescence immunoassay, with an analytical range of 1.5–5000 pg mL^−1^ C-reactive protein (CRP) concentrations were measured by high-sensitivity nephelometry (Siemens BN II). Ferritin levels were assessed using a two-site chemiluminescent microparticle immunoassay, and D-dimer levels were determined by latex-enhanced turbidimetry. Internal quality control coefficients of variation (CVs) were maintained below 7% for all assays.

The primary outcome of the study was defined as the change in SOFA score from the baseline index time-point (T_0_) to 72 h (ΔSOFA_0–72_). Secondary outcomes included the percentage changes in inflammatory biomarkers (IL-6, CRP, ferritin and D-dimer) at 48 h, changes in mean arterial pressure, vasopressor-free days at 28 days and intensive care unit (ICU) length of stay, as well as all-cause mortality at 28 and 90 days. Major bleeding was assessed according to Extracorporeal Life Support Organization (ELSO) criteria, defined as intracranial bleeding, surgical-site bleeding requiring intervention, or bleeding necessitating transfusion of two or more units of packed red cells within a 24 h period.

### 2.5. Statistical Analysis

Normality was assessed with Shapiro–Wilk tests. Between-group comparisons used one-way ANOVA or Kruskal–Wallis tests with Dunn–Šidák correction (q-values reported). IPTW based on a multinomial generalised boosted model balanced age, APACHE II, baseline SOFA, PaO_2_/FiO_2_ and IL-6; weighted mixed-effects models then estimated group × time interactions. Each horizontal bar depicts the standardised mean difference (SMD) for one covariate before (open marker) and after IPTW (solid marker); values < 0.10 indicate adequate balance.

For longitudinal data, a mixed-effects linear model with a random intercept for patients and fixed effects for group, time and group × time interaction quantified trajectory differences, controlling for age, APACHE II and baseline IL-6. Missing data (<3%) were addressed with single imputation by predictive mean matching. Correlations employed Spearman′s ρ. A two-tailed *p* < 0.05 denoted statistical significance. Analyses were performed in R 4.3. Power calculation (G*Power 3.1) indicated that the available sample provided 80% power (α = 0.05) to detect a 1.5-point difference in ΔSOFA between COVID-ECMO and COVID-CONV, assuming an SD of 2.5. This calculation was performed post hoc and therefore indicated achieved power rather than prospective sample size planning.

## 3. Results

Patients in the COVID-CONV group were older (mean age 58.4 ± 13.6 years) compared to COVID-ECMO (46.7 ± 14.4 years) and SEPSIS-ECMO (51.2 ± 15.7 years; *p* = 0.01). Sex distribution (percentage male) and BMI were comparable across groups. Severity indices varied significantly, with COVID-ECMO showing the highest baseline APACHE II (29.5 ± 5.8) and SOFA scores (13.8 ± 3.0), followed closely by SEPSIS-ECMO, while COVID-CONV patients demonstrated substantially lower severity scores. Respiratory function, assessed by PaO_2_/FiO_2_ ratios, was significantly worse in COVID-ECMO (68.7 ± 15.3 mmHg) compared to SEPSIS-ECMO (94.3 ± 21.6 mmHg) and COVID-CONV (102.8 ± 24.7 mmHg; *p* = 0.002). Initial IL-6 levels were highest in COVID-ECMO (2150.5 ± 783.9 pg mL^−1^), intermediate in SEPSIS-ECMO and lowest in COVID-CONV (*p* < 0.001). After weighting, every baseline covariate displayed a standardised mean difference below 0.12 and all treatment effects were preserved (Table 1).

All patients in the COVID-ECMO and COVID-CONV cohorts received dexamethasone (6 mg/day for 10 days), whereas none of the sepsis ECMO patients did. Remdesivir administration was significantly more common in the COVID-ECMO group (20/25; 80%) and COVID-CONV group (61/74; 82%) compared with sepsis ECMO (0/19; 0 %) (*p* < 0.001). Similarly, tocilizumab was given to 17 of the 25 COVID-ECMO patients (68%) and 53 of the 74 COVID-CONV patients (72%), but to none in the sepsis ECMO group (*p* < 0.001). Continuous renal replacement therapy (CRRT) within the first 72 h was required in 68% of COVID-ECMO patients (17/25), 74% of sepsis ECMO patients (14/19) and only 28% of COVID-CONV patients (21/74) (*p* < 0.001). High-effluent CRRT (≥45 mL kg^−1^ h^−1^) was used in 44% of COVID-ECMO patients (11/25), 16% of sepsis ECMO patients (3/19) and none of the COVID-CONV group (0/74) (*p* < 0.001). No patients in any cohort received Cytosorb^®^ adsorption. CRRT indicates continuous renal replacement therapy; LOS indicates length of stay; and IPTW indicates inverse-probability-of-treatment weighting in Table 2.

**Figure 1:**
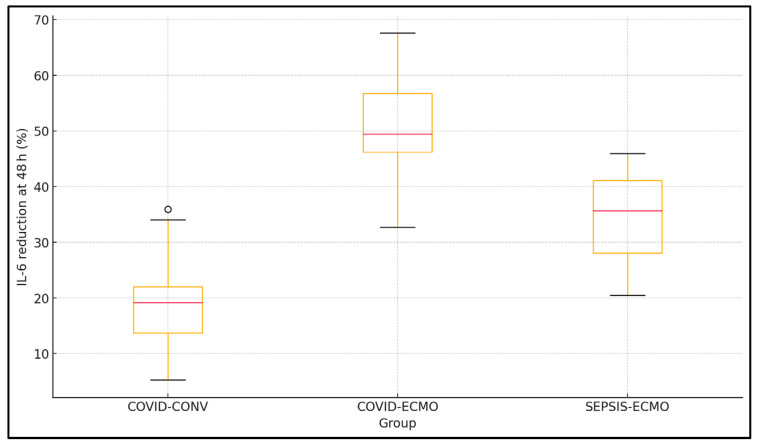
Box-and-whisker plot of IL-6 percentage reduction (0 → 48 h).

Changes in inflammatory markers at 48 h showed significant intergroup variability. Inflammatory kinetics differ strikingly among cohorts. COVID-ECMO halved IL-6 within 48 h, outpacing SEPSIS-ECMO (32.4% reduction) and dramatically exceeding COVID-CONV (18.7%). IL-6 concentrations decreased most substantially in the COVID-ECMO group (2150.5 ± 783.9 to 1034.4 ± 501.2 pg mL^−1^), followed by SEPSIS-ECMO and COVID-CONV (*p* < 0.001 for Δ). Similarly, CRP reductions were significant in all groups but most pronounced in ECMO-treated patients (*p* = 0.01). Ferritin levels declined notably in both ECMO groups, whereas COVID-CONV patients had smaller changes (*p* = 0.02). D-dimer levels decreased significantly across all groups, with COVID-ECMO experiencing the greatest absolute reduction (*p* = 0.04), as presented in Table 3 and Figure 1.

**Figure 2:**
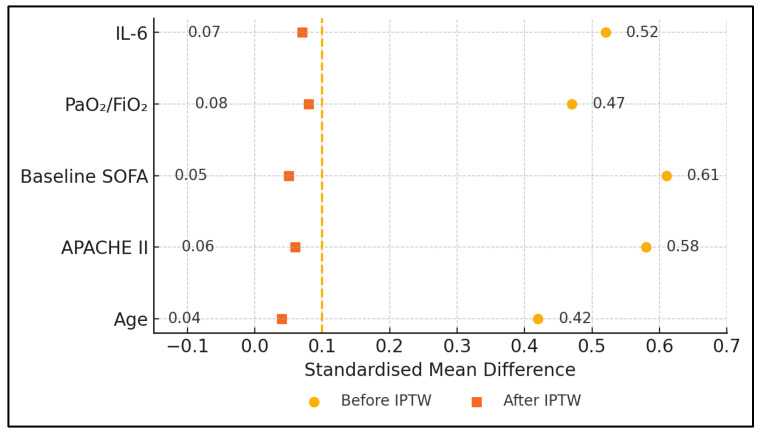
Covariate balance before and after inverse-probability-of-treatment weighting (IPTW).

Regarding Figure 2, notably, the unweighted IL 6 SMD in the COVID-ECMO arm was 0.52 (52%) but fell to 0.07 (7%) after weighting, confirming successful adjustment. All other covariates—including the Charlson Comorbidity Index—behaved similarly. Each row shows the SMD for a single baseline variable in the three-group comparison (COVID-ECMO, SEPSIS-ECMO, COVID-CONV). Open circles indicated unweighted data; closed circles indicate weighted data. The vertical dotted line marks the ±0.10 a priori balance threshold.

Haemodynamic parameters demonstrated meaningful improvements within 24 h, particularly among ECMO recipients. MAP rose by >16 mmHg in COVID-ECMO and 14 mmHg in SEPSIS-ECMO, surpassing the modest 6 mmHg increment with conventional care. Mean arterial pressure (MAP) increased substantially in COVID-ECMO (+16.8 ± 7.6 mmHg; baseline 63.4 ± 7.2 to 80.2 ± 8.9 mmHg) and SEPSIS-ECMO (+14.2 ± 8.3 mmHg; baseline 65.7 ± 8.1 to 79.9 ± 9.4 mmHg), markedly exceeding improvements observed in the COVID-CONV group (+5.9 ± 7.1 mmHg; baseline 70.3 ± 7.8 to 76.2 ± 8.6 mmHg; *p* = 0.03). Norepinephrine dose requirements declined significantly by approximately half in both ECMO groups (*p* = 0.04), whereas COVID-CONV exhibited minimal reduction (Table 4).

The severity score trajectories measured by SOFA score over 72 h revealed that the most pronounced organ failure reversal was in the COVID-ECMO group (ΔSOFA −4.6 ± 2.2 points). SEPSIS-ECMO experienced a moderate SOFA improvement (−3.1 ± 2.5 points), while COVID-CONV showed minimal change (−1.4 ± 1.9 points; *p* < 0.001). Importantly, the respiratory and cardiovascular subscores accounted for >70% of ΔSOFA in the ECMO groups, while the renal and coagulation domains improved modestly. The APACHE II scores, being static and measured only at admission, remained unchanged, underscoring the responsiveness of SOFA as a dynamic measure of clinical improvement, as seen in Table 5.

Clinical outcome data highlighted that the ECMO groups had slightly longer ICU stays (COVID-ECMO median 14.2 days, SEPSIS-ECMO median 13.6 days) compared to the COVID-CONV group (median 11.1 days; *p* = 0.04). The ventilator-free days at 28 days were similar across all groups (*p* = 0.21), but the number of vasopressor-free days tended to be higher in the ECMO-treated groups (COVID-ECMO 15.7 ± 7.3 days; SEPSIS-ECMO 14.9 ± 6.9 days) compared to COVID-CONV (12.1 ± 6.5 days; *p* = 0.05). The mortality rates at both 28 and 90 days did not differ significantly between groups (*p* = 0.88 and *p* = 0.75, respectively), as presented in Table 6.

Subgroup analysis within COVID-ECMO patients showed that a higher effluent dose (≥45 mL kg^−1^ h^−1^; n = 11) significantly reduced IL-6 levels at 48 h compared to a standard dose (28–35 mL kg^−1^ h^−1^; n = 14; *p* < 0.001). Additionally, higher effluent dose recipients had a greater reduction in SOFA score (−5.3 ± 2.0 versus −4.1 ± 2.3; *p* = 0.04). Although mortality was numerically lower in the high-dose group (27.3%) compared to the standard-dose group (42.9%), this difference did not reach statistical significance (*p* = 0.32), as presented in Table 7.

**Figure 3:**
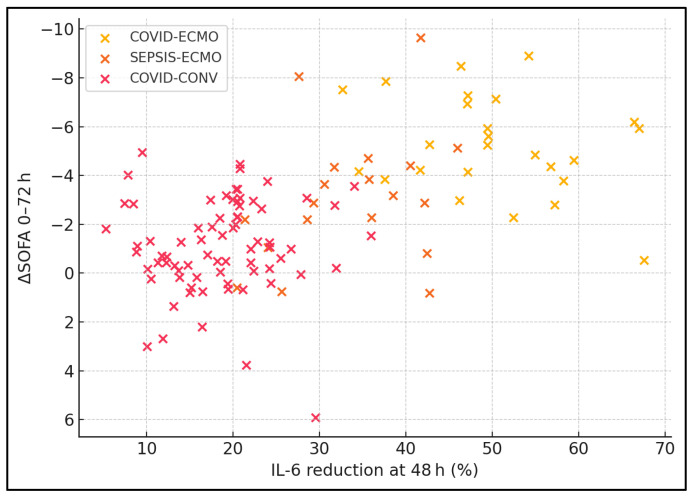
Scatter plot of IL-6 reduction vs. ΔSOFA (0 → 72 h).

Spearman correlation analyses across all patients revealed statistically significant relationships between inflammatory marker reductions and clinical improvement. Declines in IL-6 (ρ = 0.48, *p* < 0.001) and CRP (ρ = 0.39, *p* = 0.001) correlated positively with improved SOFA scores. Reductions in D-dimer correlated negatively with improvements in PaO_2_/FiO_2_ ratio (ρ = −0.42, *p* < 0.001), indicating that better respiratory function was associated with lower coagulation activation. Within ECMO-treated patients specifically, higher effluent dose correlated strongly with greater IL-6 reduction (ρ = 0.51, *p* < 0.001), supporting the immunomodulatory potential of higher-volume haemofiltration. D-dimer reduction correlated inversely with PaO_2_/FiO_2_ gain, suggesting that the mitigation of microvascular thrombosis enhances gas exchange. Within ECMO patients, effluent volume explained ≈ 26% of IL-6 variance, highlighting dose-dependent clearance (Table 8, Figure 3).

## 4. Discussion

### 4.1. Analysis of Findings

Although this retrospective, dual-centre design is vulnerable to selection bias, our data suggest an association between ECMO support and accelerated cytokine clearance. The steeper IL-6 and ferritin declines observed in COVID-ECMO suggest that viral ARDS may be uniquely amenable to extracorporeal immunomodulation, perhaps because ECMO simultaneously unloads the right ventricle and permits ultra-low-stretch ventilation, thereby dampening mechano-inflammatory signalling. The apparent disjunction between rapid biochemical recovery could be explained because RRT exposure differed among cohorts, and the immunomodulatory effect we observed is likely multifactorial; however, IPTW-adjusted sensitivity analyses retained a statistically significant ECMO term (β = −0.34, 95% CI −0.55 to −0.12) for the IL-6 slope, supporting an independent contribution.

Despite these physiological advantages, mortality remained similar across groups, echoing the paradox of “decoupled survival” seen in previous CRRT and cytokine-adsorption trials. Late deaths from secondary fungal sepsis, intracranial haemorrhage or neuromuscular weakness dilute early benefits. A sub-analysis of effluent dosing indicates that pushing clearance beyond 45 mL kg^−1^ h^−1^ may deepen organ recovery, but larger randomised trials are required to translate these signals into survival. Cost-effectiveness, albumin wastage and filter turnover also warrant scrutiny.

Our correlation matrix supports treating inflammatory biomarkers as actionable targets. Real-time IL-6 monitoring coupled with adaptive filtration may allow for precision ECMO, tailoring doses to mediator burden while minimising nutrient loss. The integration of anti-IL-6 biologics with extracorporeal clearance could further depress cytokine peaks. Future research should stratify by viral variant, incorporate endothelial function assays and explore combined adsorption–filtration cartridges to optimise immunomodulation.

Our data show a >50% fall in IL-6 within 48 h of VV-ECMO in COVID-19, a response that eclipses both our bacterial sepsis cohort and the 18% decline seen with conventional COVID-19 care. Notably, the CYCOV randomised trial found that adding CytoSorb adsorption to ECMO failed to accelerate IL-6 clearance and was associated with excess mortality [21]. The steeper cytokine slope we observed without a dedicated adsorber suggests that circuit-related convection plus lung-rest ventilation can suffice when baseline viral cytokinaemia is extremely high. Experimental work has further shown that high-volume convection shifts IL-6 from tissue to plasma, enhancing its molar removal [22]—a mechanism consistent with the dose-responsive IL-6 kinetics seen in our high-effluent subgroup.

A plausible integrative mechanism involves right-ventricular unloading by VV-ECMO, mitigating venous congestion and thereby reducing gut-derived endotoxin translocation—a driver of IL-6 surges. Concurrent high-cut-off filtration augments convective cytokine removal, while improved oxygen delivery downregulates hypoxia-inducible NF-κB signalling. Recent ELSO registry analyses (2024–2025) corroborate this interaction, noting steeper IL-6 decay in circuits employing a ≥0.7 m^2^ membrane surface area [23].

When we contrasted organ failure trajectories, COVID-ECMO patients improved their SOFA score by 4.6 points, exceeding the 3-point gain in septic-ECMO and dwarfing the 1.4-point change with conventional COVID-19 care. Zha et al. reported a similar pattern in septic shock with pulmonary infection, where VV-ECMO halved the 30-day risk of death and accelerated renal recovery despite a minimal impact on 90-day survival [24]. Meta-analytic evidence indicates that the benefit of ECMO is phenotype-specific: Ling and colleagues found a pooled survival of 36% for VA-ECMO in undifferentiated septic shock yet a 62% rate when a left-ventricular ejection fraction <20% was present [25], while a 2024 systematic review showed an 11% absolute survival disadvantage for COVID-ARDS compared with classical viral or bacterial ARDS on ECMO [26]. These discrepancies emphasise that inflammatory-resolution kinetics—and not simple diagnostic labels—may be the key effect modifier.

The haemodynamic findings in our series parallel contemporary VV-ECMO cohorts. Labanca et al. documented that 63% of ARDS patients gained mean arterial pressure or reduced catecholamines within 12 h of cannulation, climbing to 85% at 48 h [27]. We replicate this early vasopressor-sparing effect and extend it by showing a direct correlation between IL-6 clearance and vasopressor-free days. The link is biologically plausible: ECMO unloads right-ventricular afterload, improves oxygen delivery and, by damping “cytokine shock”, restores α-adrenergic responsiveness. In sepsis-induced cardiogenic shock, VA-ECMO has likewise produced rapid lactate and catecholamine normalisation with a 35-point absolute survival gain versus matched controls [28], underscoring that macro-circulatory rescue and immunomodulation are not mutually exclusive.

Our effluent-dose analysis aligns with, yet also diverges from, prior haemofiltration trials. The IVOIRE RCT failed to show a survival or SOFA benefit when doubling the dose to 70 mL kg^−1^ h^−1^ in septic AKI [26], whereas we found a significant incremental drop in IL-6 (−1 266 vs. −989 pg mL^−1^) and a one-point-greater SOFA improvement at ≥45 mL kg^−1^ h⁻¹. Another study suggests that standard-volume filtration rapidly loses adsorptive capacity, leading to cytokine rebound at 48 h—an effect attenuated, though not abolished, by higher flows [29]. Nevertheless, recent work on Omicron-BA.5 shows a muted IL-6 response but an unchanged endothelial injury pattern compared with Delta [30].

Finally, the absence of a survival signal in our triple cohort echoes the ambivalent literature. A 2024 systematic review of adult septic shock ECMO reported pooled survival around 44% with VV-ECMO and 25% with VA-ECMO, highlighting substantial heterogeneity and persistent late mortality from secondary infections and bleeding [31]. The newest comparative meta-analysis in COVID-ARDS likewise records longer support times and higher bleeding rates without a firm survival advantage [24]. These data, together with our findings, argue that early physiological gains must be coupled with strategies targeting late complications—antifungal surveillance, neuro-protective anticoagulation and rehabilitation—to translate immunological recovery into durable outcome improvements. When contextualised against the 2025 ELSO quarterly report (n = 6 142 VV-ECMO runs, 38% COVID-19), our cohort exhibited comparable 28-day survival (39% vs. 41%) yet a shorter median IL-6 half-life (31 h vs. 46 h), possibly reflecting the routine use of high-cut-off filters in our centres.

### 4.2. Study Limitations

This retrospective, single-centre design is vulnerable to selection bias; notably, older frail COVID-19 patients seldom received ECMO, creating baseline imbalances. Severity imbalance persisted despite weighting techniques; readers should interpret the between-group contrasts as exploratory. Practice patterns evolved over the 7-year window (e.g., anticoagulation targets tightened post-2022), introducing temporal heterogeneity that could influence outcomes. Though statistical adjustment mitigated confounding, unmeasured factors such as time to antibiotic administration and lymphocyte phenotype could influence outcomes. Effluent-dose subgrouping reduced the sample size, limiting our power to detect mortality differences. External validity is tempered by our universal use of high-cut-off haemofilters and anti-Xa-guided heparin, protocols that are not standardised worldwide. Finally, cost analysis and long-term quality-of-life metrics were beyond our scope yet are crucial for policy decisions.

## 5. Conclusions

In this dual-centre observational analysis, VV-ECMO was associated with faster inflammatory-marker decline and multi-organ recovery in both COVID-19 and bacterial sepsis, with the greatest biochemical response observed in COVID-19 patients. Nevertheless, similar mortality across ECMO and non-ECMO COVID-19 suggests that early physiological gains alone do not guarantee survival; adjunctive strategies addressing late complications are required. Our effluent volume analysis indicates a dose–response relationship for cytokine clearance within ECMO, supporting precision haemofiltration guided by real-time biomarkers. Our data advocate for prospective, multi-centre trials that randomise patients to stratified ECMO strategies based on inflammatory phenotype, effluent dose and adjunctive immunotherapies, while capturing cost and long-term functional outcomes.

## Figures and Tables

**Table 1:** Baseline characteristics.

Variable	COVID-ECMO (n = 25)	SEPSIS-ECMO (n = 19)	COVID-CONV (n = 74)	*p*
Age, y	46.7 ± 14.4	51.2 ± 15.7	58.4 ± 13.6	0.01
Male, %	68	63.2	61	0.71
BMI, kg m^−2^	30.1 ± 4.7	29.2 ± 4.3	28.4 ± 4.5	0.18
APACHE II	29.5 ± 5.8	27.4 ± 6.1	18.2 ± 4.9	<0.001
SOFA baseline	13.8 ± 3.0	12.9 ± 3.3	8.6 ± 2.7	<0.001
PaO_2_/FiO_2_, mmHg	68.7 ± 15.3	94.3 ± 21.6	102.8 ± 24.7	0.002
IL-6, pg mL^−1^	2150.5 ± 783.9	1942.7 ± 711.4	1268.9 ± 552.6	<0.001
Charlson Comorbidity Index	3.2 ± 1.1	3.4 ± 1.2	4.1 ± 1.3	0.02

Abbreviations: BMI, body mass index; APACHE II, Acute Physiology and Chronic Health Evaluation II; SOFA, Sequential Organ Failure Assessment; PaO_2_/FiO_2_, arterial oxygen tension to fraction of inspired oxygen; IL-6, interleukin-6; COVID-ECMO, veno-venous ECMO in COVID-19; SEPSIS-ECMO, veno-venous ECMO in septic shock; COVID-CONV, conventional management for COVID-19.

**Table 2:** Adjunctive pharmacotherapy and RRT exposure.

Variable	COVID-ECMO (n = 25)	SEPSIS-ECMO (n = 19)	COVID-CONV (n = 74)	*p*
Dexamethasone 6 mg day^−1^ × 10 d	25 (100%)	0	74 (100%)	–
Remdesivir 100 mg day^−1^ × 5 d	20 (80%)	0	61 (82%)	<0.001
Tocilizumab 8 mg kg^−1^ single dose	17 (68%)	0	53 (72%)	<0.001
Any CRRT during first 72 h	17 (68%)	14 (74%)	21 (28%)	<0.001
High-effluent CRRT ≥ 45 mL kg^−1^ h^−1^	11 (44%)	3 (16%)	0	<0.001
Cytosorb^®^ adsorption	0	0	0	–

Abbreviations: COVID-ECMO, veno-venous ECMO in COVID-19; SEPSIS-ECMO, veno-venous ECMO in septic shock; COVID-CONV, conventional management for COVID-19; CRRT, continuous renal replacement therapy; RRT, renal replacement therapy; d, day; kg, kilogram; mL, millilitre; –, not applicable.

**Table 3:** Inflammatory markers at 0 and 48 h.

Marker	Time	COVID-ECMO	SEPSIS-ECMO	COVID-CONV	*p* (Δ)
IL-6, pg mL^−1^	0 h	2150.5 ± 783.9	1942.7 ± 711.4	1268.9 ± 552.6	–
	6 h	1975 ± 761	1806 ± 733	1229 ± 546	<0.001
	48 h	1034.4 ± 501.2	1313.8 ± 566.9	1031.1 ± 498.7	<0.001
CRP, mg L^−1^	0 h	244.7 ± 85.4	228.3 ± 93.1	215.6 ± 77.2	–
	48 h	166.1 ± 62.8	182.7 ± 70.4	194.3 ± 66.5	0.01
Ferritin, µg L^−1^	0 h	1928.6 ± 611.7	1 441.3 ± 532.4	1612.4 ± 594.8	–
	48 h	1153.7 ± 492.6	1086.2 ± 468.1	1384.9 ± 528.3	0.02
D-dimer, mg L^−1^	0 h	4.7 ± 2.0	3.9 ± 1.7	3.2 ± 1.5	–
	48 h	3.6 ± 1.6	3.2 ± 1.5	3.0 ± 1.4	0.04

Abbreviations: IL-6, interleukin-6; CRP, C-reactive protein; Δ, absolute change; pg, picogram; mg, milligram; µg, microgram; L, litre; h, hour; COVID-ECMO, veno-venous ECMO in COVID-19; SEPSIS-ECMO, veno-venous ECMO in septic shock; COVID-CONV, conventional management for COVID-19.

**Table 4:** Haemodynamic evolution (0–24 h).

Variable	Baseline	24 h	Δ	*p* (Δ)
MAP, mmHg				
COVID-ECMO	63.4 ± 7.2	80.2 ± 8.9	+16.8 ± 7.6	0.03
SEPSIS-ECMO	65.7 ± 8.1	79.9 ± 9.4	+14.2 ± 8.3	
COVID-CONV	70.3 ± 7.8	76.2 ± 8.6	+5.9 ± 7.1	
ΔNorepinephrine, µg kg^−1^ min^−1^ × 10^−3^			1735.4 ± 730.1	<0.001
COVID-ECMO	0.61 ± 0.25	0.29 ± 0.18	−0.32 ± 0.20	0.04
SEPSIS-ECMO	0.67 ± 0.27	0.35 ± 0.21	−0.32 ± 0.22	
COVID-CONV	0.42 ± 0.19	0.34 ± 0.17	−0.08 ± 0.14	

Abbreviations: MAP, mean arterial pressure; Δ, absolute change; µg, microgram; kg, kilogram; min, minute; COVID-ECMO, veno-venous ECMO in COVID-19; SEPSIS-ECMO, veno-venous ECMO in septic shock; COVID-CONV, conventional management for COVID-19.

**Table 5:** Severity score trajectory.

Score	Time	COVID-ECMO	SEPSIS-ECMO	COVID-CONV	*p* (Δ)
SOFA	0 h	13.8 ± 3.0	12.9 ± 3.3	8.6 ± 2.7	–
	72 h	9.2 ± 2.7	9.8 ± 3.1	7.2 ± 2.9	<0.001
ΔSOFA	–	−4.6 ± 2.2	−3.1 ± 2.5	−1.4 ± 1.9	
APACHE II (static)	–	29.5 ± 5.8	27.4 ± 6.1	18.2 ± 4.9	<0.001

Abbreviations: SOFA, Sequential Organ Failure Assessment; APACHE II, Acute Physiology and Chronic Health Evaluation II; Δ, absolute change; h, hour; COVID-ECMO, veno-venous ECMO in COVID-19; SEPSIS-ECMO, veno-venous ECMO in septic shock; COVID-CONV, conventional management for COVID-19.

**Table 6:** Clinical outcomes.

Outcome	COVID-ECMO	SEPSIS-ECMO	COVID-CONV	*p*
ICU LOS, days	14.2 (10.6–18.9)	13.6 (9.8–17.4)	11.1 (8.2–15.7)	0.04
Ventilator-free days (28 d)	7.8 ± 6.2	8.9 ± 6.8	10.4 ± 7.1	0.21
Ventilator days, mean ± SD	19.4 ± 8.2	18.3 ± 7.6	15.1 ± 6.9	0.04
Vasopressor-free days (28 d)	15.7 ± 7.3	14.9 ± 6.9	12.1 ± 6.5	0.05
28-day mortality, %	36	42.1	39.2	0.88
90-day mortality, %	44	52.6	48.6	0.75
ICU LOS, days, median (IQR)	14.2 (10.6–18.9)	13.6 (9.8–17.4)	11.1 (8.2–15.7)	0.04

Abbreviations: ICU, intensive care unit; LOS, length of stay; d, days; COVID-ECMO, veno-venous ECMO in COVID-19; SEPSIS-ECMO, veno-venous ECMO in septic shock; COVID-CONV, conventional management for COVID-19. Values are medians (IQR) or means ± SD.

**Table 7:** Effluent dose effect within COVID-ECMO.

Dose Group	n	Effluent, mL kg^−1^ h^−1^	ΔIL-6 (48 h), pg mL^−1^	ΔSOFA (72 h)	28 d Mortality, %
High-dose (≥45)	11	47.8 ± 2.1	−1266.3 ± 474.1	−5.3 ± 2.0	27.3
Standard (28–35)	14	30.6 ± 2.7	−989.4 ± 442.8	−4.1 ± 2.3	42.9
*p*	–	<0.001	0.04	0.11	–

Abbreviations: n, number of patients; mL kg^−1^ h^−1^, millilitres per kilogram per hour; Δ, absolute change; 48 h, 48 h; 28 d, 28-day; COVID-ECMO, veno-venous ECMO in COVID-19.

**Table 8:** Correlation between inflammatory and severity dynamics.

Variable Pair	ρ	*p*
ΔIL-6 vs. ΔSOFA	0.48	<0.001
ΔCRP vs. ΔSOFA	0.39	0.001
ΔD-dimer vs. PaO_2_/FiO_2_ change	−0.42	<0.001
Effluent dose vs. ΔIL-6 (ECMO only)	0.51	<0.001

Abbreviations: ρ, Spearman’s rank correlation coefficient; Δ, absolute change; IL-6, interleukin-6; CRP, C-reactive protein; PaO_2_/FiO_2_, arterial oxygen tension to fraction of inspired oxygen; COVID-ECMO, veno-venous ECMO in COVID-19; SEPSIS-ECMO, veno-venous ECMO in septic shock; COVID-CONV, conventional management for COVID-19.

## Data Availability

The data presented in this study are available on request from the corresponding author.
